# Prognostic effect of adjuvant chemoradiotherapy for patients with gastric cancer: an updated evidence of randomized controlled trials

**DOI:** 10.18632/oncotarget.21983

**Published:** 2017-10-24

**Authors:** Mei-Juan Wang, Chao Li, Yue Sun, Fu-Jun Shen, Chun-Bin Wang

**Affiliations:** ^1^ Department of Oncology, Yancheng Hospital Affiliated to Medical College of Southeast University and The Third People’s Hospital of Yancheng, Yancheng, Jiangsu, 224001, China

**Keywords:** chemoradiotherapy, gastric cancer, survival, prognosis, randomized controlled trial

## Abstract

The prognostic effect of chemoradiotherapy in gastric cancer has been evaluated for decades while the results are still in debate and heterogeneous.

We thus comprehensively updated the evidence through systematic review and meta-analysis to evaluate chemoradiotherapy in gastric cancer to determine its effect. Pubmed, EMBASE, and Cochrane Library from the earliest possible year to April 2017 were searched. Randomised controlled trials (RCTs) that assessed the effects of combined chemoradiotherapy for patients with gastric cancer compared with that of single chemotherapy were included. The main outcome measure was 5-year overall survival (OS) and the second was disease-free survival (DFS) or recurrence-free survival (RFS).

Fifteen RCTs involving 3347 patients were included into this meta-analysis. Compared with single chemotherapy, the relative risk (RR) for 5-year OS for chemoradiotherapy was 1.05 (95% CI 0.88 to 1.25), with moderate heterogeneity across eligible trials (*I*^2^ = 55.7%, *p* = 0.016). Subgroup analyses and sensitivity analyses confirmed the consistent findings. We found that significant survival benefit for 5-year DFS/RFS for chemoradiotherapy over single chemotherapy (RR 0.89 95% CI 0.81 to 0.98) for patients with gastric cancer. This updated meta-analysis does not provide strong evidence for a 5-year survival benefit of chemoradiotherapy over chemotherapy alone in patients with gastric cancer. A clear advantage of chemoradiotherapy over chemotherapy has not been established. Further larger RCTs should be conducted to determine its true effect.

## INTRODUCTION

Gastric cancer remains one of the leading causes of cancer-related death worldwide, with approximately 950,000 new diagnoses each year and 720,000 deaths in 2012 [1]. The optimal therapeutic option for patients with resectable gastric cancer is surgical intervention. Adjuvant therapies were given mainly to improve postoperative survival in patients who have received R0 resection of locally advanced gastric cancer, with an estimated increase of 5-year overall survival (OS) by 10–15% [2]. However, no consensus about the optimal treatment strategy has been reached. It has been shown that patients with gastric cancer receiving postoperative fluoropyrimidine-based and platinum-based chemotherapy gain survival benefits [3, 4]. In recent years, several clinical trials have found a potential advantage of adjuvant chemoradiation over chemotherapy [5–7]. The US 0116 trial found that compared with surgery, chemoradiotherapy could significantly reduce mortality and risk of tumor recurrence [8, 9]. However, the ARTIST trial conducted in South Korea showed that for patients with gastric cancer who underwent D2 lymph-node dissection, chemoradiotherapy to chemotherapy did not add survival benefits in terms of disease-free survival (DFS) or OS [10]. Therefore, the prognostic effects of adjuvant chemoradiotherapy for patients with gastric cancer remain uncertain. We therefore performed an updated meta-analysis to reassess the prognostic value for chemoradiotherapy for gastric cancer patients receiving surgery.

## RESULTS

### Study characteristics

**Figure 1:**
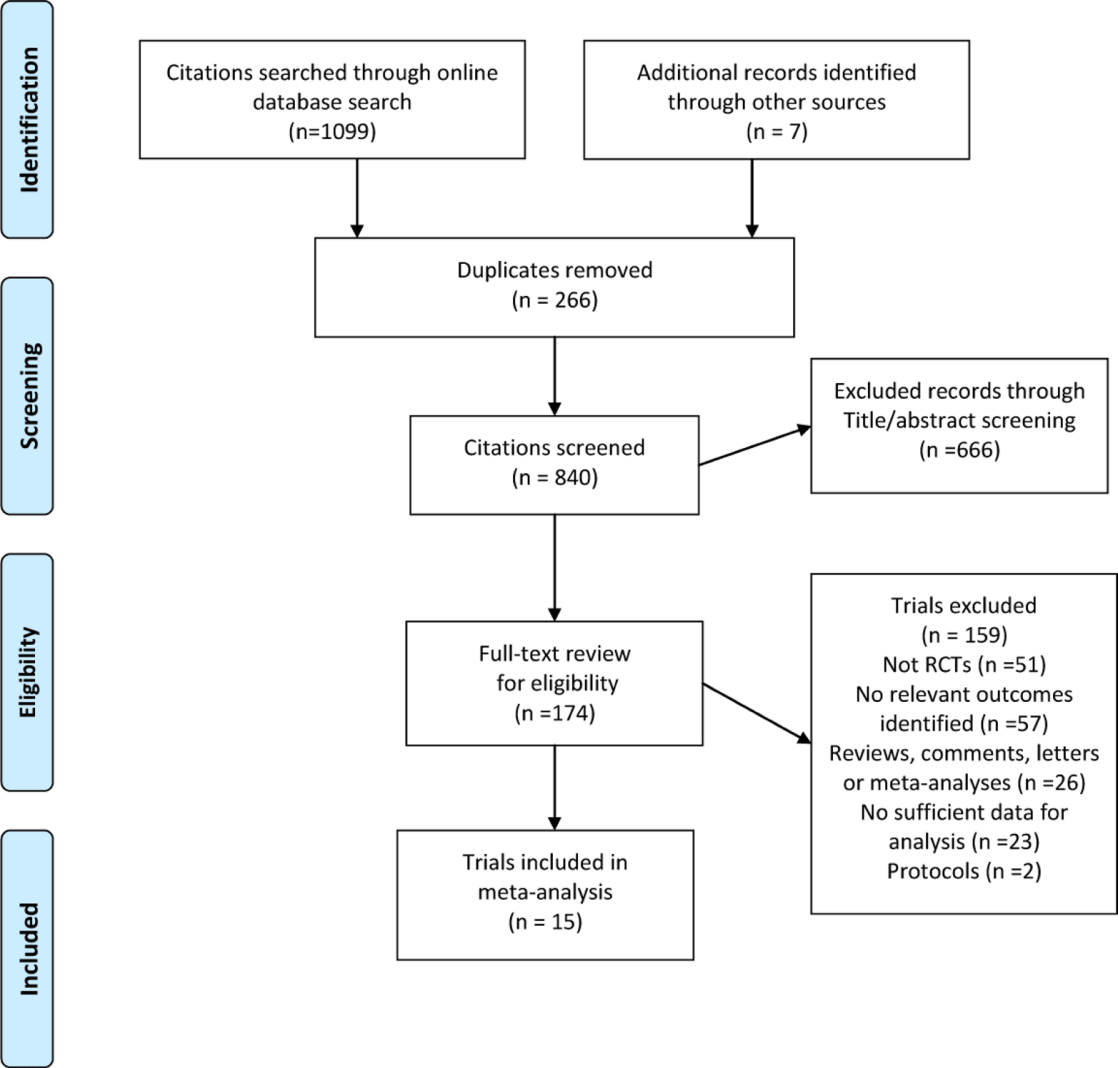
Flow chart for the process of identifying trials included in and excluded from the systematic review

We identified 1099 records from the database search and seven additional records from other sources (Figure 1). After title or abstract screening, 174 relevant records met our inclusion criteria, and their full texts were assessed. Of these, 15 RCTs contained at least one common outcome with sufficient data [3–7, 11–20], leaving 10 and six eligible trials for 5-year OS and DFS/recurrence-free survival (RFS) analysis, respectively. The trials were published between 1979 to 2015 as full articles. There were 1575 patients in the experiment arm (chemoradiotherapy group) and 1541 patients in the control arm (chemotherapy group), with a range of sample size from 61 to 559. Three trials were performed in USA [6, 15, 16], five in Europe [11, 13, 17–19], six in Asia [3–5, 7, 14, 20] and one in Africa [12]. The median follow-up ranged from 24 to 128 months. Almost all the patients from the included trials received surgery. The total dose of radiotherapy was 45Gy in 8 of the 15 trials and 20Gy in 4 trials. Ten trials reported 5-year OS and 6 reported 5-year DFS or RFS (Table 1). The methodological quality of each trial eligible for this meta-analysis is summarized in Supplementary Table 1.

**Table 1:** Major features of the included randomized controlled trials

First author	Publication year	Country	Inclusion period	Mean/median age(years): (Exp. versus Control)	Tumor stage	Median follow up period (months)	Total sample size	No.CRT	No.CT	Lymphadenectomy	Mean/median No. of dissected LNs(Exp. versus Control)	Treatment regimen	Total RT dose (Gy)	Primary endpoints
Yu	2012	China	2006–2007	56 versus 57	II	36	68	34	34	D1,D2	NR	S+CRT/S+CT	45	3-year OS; 3-year DFS
Smalley	2012	USA	1991–1998	NR	I-IV	123.6	559	282	277	D0,D1,D2	NR	S+CRT/S	45	10-year OS; 10-year RFS
Kim	2012	Korea	2002–2006	≤ 60 (80.4) versus ≤ 60 (68.2)	III-IV	86.7 (60.3–116.5)	90	46	44	D2	46.5 (21–93) versus 41 (22–129)	S+CRT/S+CT	45	10-year OS;10-year RFS
Zhu	2012	China	2003–2008	56 (38–73) versus 59 (42–75)	I-IV	42.5	351	186	165	D2	NR	S+CRT/S+CT	45	5-year OS; 5-year DFS
Bamias	2010	Greece	2002–2005	63 (32–75) versus 62 (41–79)	I-IV	53.7 (0.1–77.8)	143	72	71	D0,D1,D2	14 (3–76) versus 14 (0–62)	S+CRT/S+CT	45	3-year DFS
Kwon	2010	Korea	2002–2004	56 (23–73) versus 49 (29–70)	III-IV	77.2 (24–92.8)	61	31	30	D2	NR	S+CRT/S+CT	45	5-year OS; 5-year DFS
Stahl	2009	Germany	2000–2005	56.0 versus 60.6	I-IV	45.6	126	62	64	D2	22 (5–61) versus 16 (7–38)	S+CRT/S+CT	30	3-year os
Moertel	1984	USA	1965–1974	58 (40–72) versus 56 (41–67)	NR	NR	62	39	25	NR	NR	S+CRT/S	37.5	5-year OS; 5-year RFS
Hallissey	1994	UK	1981–1986	65 (55–69) versus 63 (57–69)	II-IV	84	436	153	145	D1	NR	S+RT/S	45	5-year OS
Zhang	1998	China	1978–1989	55.8 (39–66) versus 56.1 (32–65)	I-IV	128 (89–192)	370	171	199	NR	NR	S+RT/S	40	5-year OS;10-year OS
Skoropard	2002	Russia	1974–1978	55 (25–75) versus 54 (36–71)	I-IV	240	152	77	75	D1	NR	S+RT/S	20	5-year OS;10-year OS
Skoropard	2000	Russia	1993–1998	54 (27–79) versus 55 (28–74)	NR	72	112	59	53	D1	NR	Pre RT& IORT/S	20	5-year OS; 5-year RFS
Shchepotin	1994	USA	1984–1986	55 (26–76)	II-IV	NR	293	98	100	NR	NR	S+RT/S	20	3-year OS
Dent	1979	South Africa	1974–1976	NR	NR	NR	66	35	31	NR	NR	S+CRT/S	20	3-year OS;5-year OS
Park	2015	Korea	2004–2008	56 (28–76) versus 56 (22–77)	I-IV	84	458	230	228	D2	40 (12–84) versus 40 (13–142)	S+CRT/S+CT	45	5-year OS; 5-year DFS

Abbreviations: CRT = chemoradiotherapy; CT = chemotherapy; DFS = disease-free survival; LNs = lymph nodes; NR = not report; OS = overall survival; RFS = recurrence-free survival; RT = radiotherapy; S = surgery.

### Primary endpoint: 5-year OS

**Figure 2:**
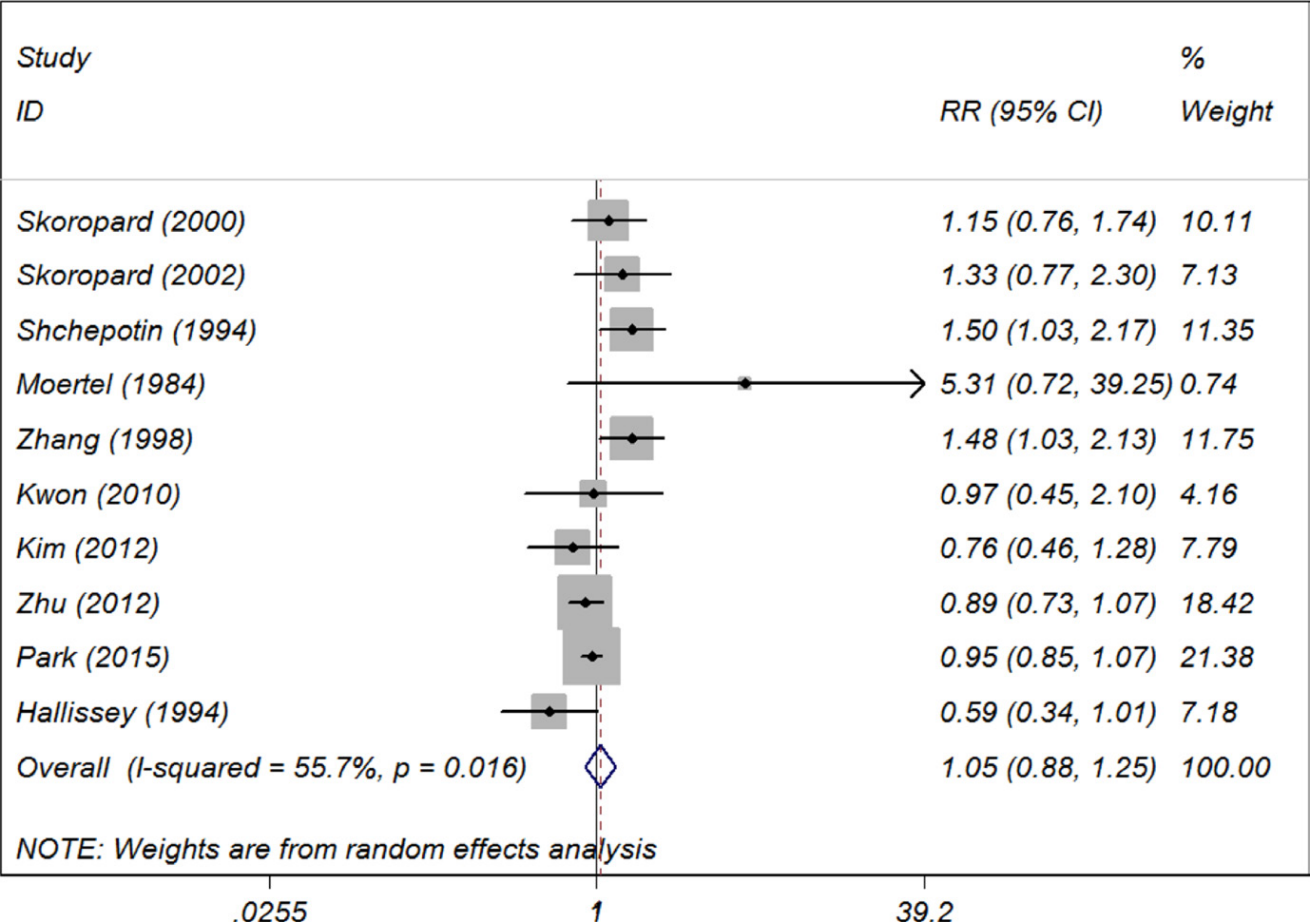
Summary estimates and 95% CIs for 5-year overall survival comparing chemotherapy alone with combined chemoradiotherapy in patients with gastric cancer Weights are from random effects analysis. Abbreviations: CI, confidence interval; RR, relative risk; W (random): Weights (random effects model).

Ten included trials provided 5-year OS data, and the relative risk (RR) and 95% CI for each study as well as the pooled RR are presented in Figure 2. The overall summary estimated RR for 5-year OS was 1. 05 (95% CI: 0.88 to 1.25). Moderate heterogeneity was revealed that *I*^2^ was 55.7% and the *P* for heterogeneity was 0.016, using a random-effect model. We also conducted subgroup analyses stratified by inclusion period, trial region, sample size, treatment regimen, total radiotherapy dose and patient follow-up period and the results indicated that some of the heterogeneity of most of the subgroups decreased significantly, but the summary estimates remained constant with no 5-year OS benefits.

### Secondary endpoint: 5-year DFS/RFS

Six trials provided 5-year DFS/RFS data and the RR and 95% CI for each study and the overall summary estimated RR was 0.89 (95% CI: 0.81 to 0.98). No heterogeneity was tested that *I*^2^ = 0% and the *P* for heterogeneity was 0.44, using a fixed-effect model.

### Publication bias and sensitivity analyses

**Figure 3:**
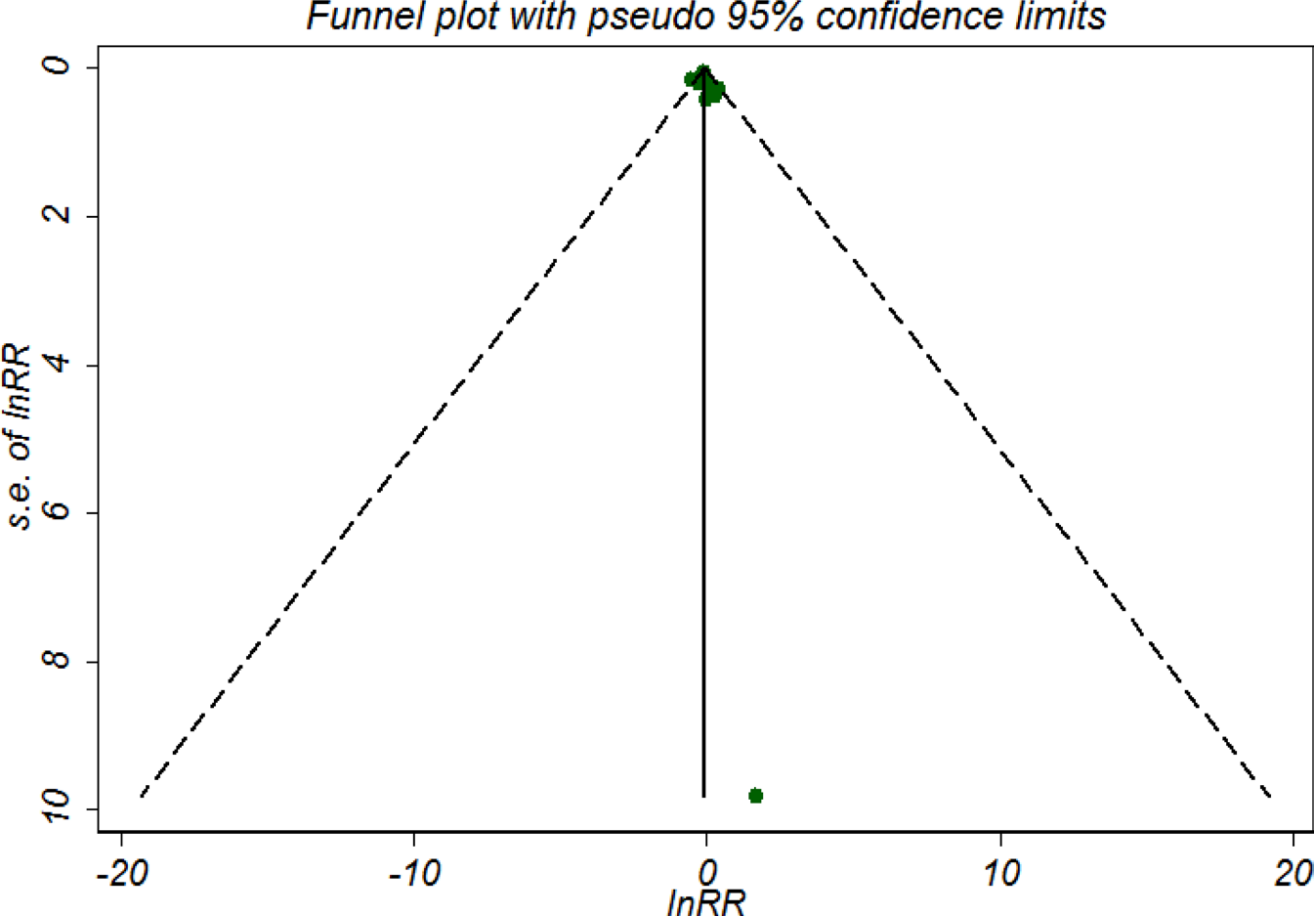
Symmetric funnel plot of trials included in meta-analysis suggesting no evidence of publication bias

For 5-year OS, the funnel plot (Figure 3) shows little evidence of asymmetry, and the results from both the Begg’s test (*P* = 0.929) and the Egger’s test (*P* = 0.247) also indicated no evidence of publication bias. Sensitivity analyses by using the trim and fill method and the ‘‘one study removed’’ procedure, the adjusted results remained unchanged. We did not test pulication bias for 5-year DFS/RFS due to limited included trials.

## DISCUSSION

This meta-analysis, based on 15 RCTs, compared survival outcomes with chemoradiotherapy versus chemotherapy alone for patients with gastric cancer receiving surgery. The findings revealed no evidence of an increased 5-year OS benefit for chemoradiotherapy compared with chemotherapy alone. However, chemoradiotherapy could provide a 5-year DFS/RFS benefit.

Several previous meta-analyses have investigated the effect of adjuvant radiotherapy for gastric cancer [21–25]. Though most of the meta-analyses yielded survival advantage of radiotherapy, some other trials did not find significant survival advantage of chemoradiotherapy over chemotherapy [11, 12, 14, 15, 17, 19]. Ours is the first one on this topic to specially address long-term survival benefits in terms of 5-year OS and DFS or RFS. The findings of our report are partly similar to five previous published meta-analyses. Dai *et al* reported that the use of chemoradiotherapy was associated with a 50% increase in 5-year DFS [25]. Others also found consistent results for postoperative radiotherapy [21, 22]. However, we did not find significant survival benefit for chemoradiotherapy in terms of 5-year OS.

5-year OS is the most frequently recorded long-term outcome in the clinical studies for gastric cancer and the most accurately measure after treatment. Although the difference between chemoradiotherapy and chemotherapy alone did not reach statistical significance for all the included trials (RR 1.05 95% CI 0.88 to 1.25), the trend toward an improved 5-year DFS/RFS was observed for the treatment of chemoradiotherapy over single chemotherapy (RR 0.89 95% CI 0.81 to 0.98). Updating the pooled results from the current included trials in a meta-analysis increases statistical power and may provide sufficient evidence to reassess the survival effect of chemoradiotherapy more reliably. The included trias were then stratified into several subgroups according to baseline features, and consistently, almost all the subgroup analyses showed that there was no statistical significant difference between chemoradiotherapy and single chemotherapy for 5-year OS (Table 2).

**Table 2:** Subgroup analyses based on some baseline characteristics of included randomized controlled trial for 5-year overall survival

	Hazard ratio	95% Confidence interval	Degree of heterogeneity (*I*^2^ statistics; %)	*P*-value	P^*^-value	No. of included Studies
**Inclusion period**						
**Before year 2000**	1.21	0.83 to 1.76	64.9	0.023	0.012	5
**After year 2000**	0.94	0.85 to 1.03	0	0.630		5
**Study region**						
**USA**	1.90	0.72 to 4.96	32.5	0.224	0.038	2
**Europe**	0.98	0.61 to 1.55	61.4	0.075		3
**Asia/Africa**	0.98	0.83 to 1.15	43.4	0.132		5
**Sample size**						
**<300**	1.19	0.91 to 1.55	28.1	0.224	0.047	6
**≥300**	0.96	0.77 to 1.19	68.1	0.024		4
**Treatment regimen**						
**S+CRT/S+CT**	1.05	0.88 to 1.02	0	0.82	0.007	4
**S+CRT/S**	5.31	0.72 to 39.24	−	−		1
**S+RT/S**	1.19	0.88 to 1.61	57.5	0.051		5
**Total RT dose (Gy)**						
**20 Gy**	1.33	1.04 to 1.71	0.0	0.646	0.001	3
**45 Gy**	0.91	0.83 to 1.00	0.0	0.473		5
**others**	1.89	0.71 to 5.03	33.8	0.219		2
**Follow-up period (ms)**						
**<60**	0.89	0.73 to 1.07	−	−	0.026	1
**≥60**	1.01	0.82 to 1.25	47.8	0.074		7

Abbreviations: CRT = chemoradiotherapy; CT = chemotherapy; ms = months; RT = radiotherapy; S = surgery.

Note: P^*^ indicated the difference of between- subgroup heterogeneity

Although there are subtle differences between the measurement of PFS and RFS in terms of definition, we summarised these two outcome measures together in that few articles reporting these two outcomes while have more similarities. While this meta-analysis indicated that chemoradiotherapy benefit more than chemotherapy alone for DFS/RFS, we could not draw a solid conclusion as the small statistical power. More large-scale high-quality RCTs will add the evidence to confirm this association.

Several major limitations have to be addressed in this meta-analysis. Firstly, the design of the included trials varied to the different extent in the meta-analysis. For instance, the dose of radiotherapy ranged from 20–45 Gy with different timing which were not thoroughly investigated due to insufficient data from original reports. Secondly, for the nature of study level meta-analysis, we could not fully adjust some prognostic influential factors, such as tumor stage, patient age, molecular and pathological features, which could not allow us to perform related subgroup analyses. We need further well designed clinical trials to explore the best available evidence on the subject. Thirdly, other relatively short-term outcomes or adverse events are not assessed. Therefore, more multicenter well designed clinical trials are needed to further clarify the real prognostic role in specific patient profiles for gastric cancer.

There are several strengths for our meta-analysis. Firstly, thoroughly database search was conducted without language or publication date limits, to a great extent minimising the risk of missing trials. Secondly, we included the largest sample size of more than 3300 patients and conducted the most comprehensive meta-analysis regarding chemoradiotherapy and 5-year OS for gastric cancer, providing the most reliable evidence for this subject to date. Thirdly, stratified analyses have been performed based on some of the major trial features, such as study design and patient characteristics, treatment regimen and follow-up period, and the results were generally consistent and reliable. Fourthly, literature search, selection and bias assessent were done with cross-check style by two authors independently and reassessed by a senior author, guarantee the process of systematic review more objectively.

In summary, this updated meta-analysis does not provide strong evidence for a 5-year survival benefit of chemoradiotherapy over chemotherapy alone in patients with gastric cancer. A clear advantage of chemoradiotherapy over chemotherapy has not been established. Further larger RCTs should be conducted to determine its true effect. We suggest that future RCTs should be well designed for more consistent patient selection criteria, such as the consistent surgical approach and adjuvant strategy of gastric cancer, similar patient features including lymph node status and tumor histology.

## MATERIALS AND METHODS

### Search strategy

We conducted a systematic literature search of three databases including PubMed, Embase and the Cochrane Library from inception through April 2017 for all randomized controlled trials investigating the prognostic effect of combined chemoradiotherapy for patients with gastric cancer compared with that of single chemotherapy. We presented the detailed search strategies of the three databases in Supplementary materials. In summary, the following MeSH/EMTREE terms jointed with free-text words are utilized: “gastric/stomach”, “cancer*/tumo?r*/carcinom*/neoplas*/adenocarcinoma* /malignan*”, “radiotherap* OR radio-therap*”, and “randomized controlled trial/ controlled clinical trial/randomi?ed/placebo/randomly”. We also tracked the citations of the included trials and reviews identified during the selection process for additional relevant publications. Unpublished grey literature was not searched for limited data available. The searching date or language was not restricted.

### Study selection and inclusion criteria

Two investigators (MW and CL) assessed trial inclusion eligibility independently through reading the titles or abstracts identified through database search. If the citations were evaluated to be relevant, then they would be retrieved for full text reviews. Any disagreements were resolved by consensus or by a senior investigator (CW) if necessary.

Randomized controlled trials were selected eligible for inclusion if they met the following criteria: (1) randomized in trial design; (2) enrolled adults who were clinically and pathologically diagnosed gastric cancer; (3) presented prognostic data for gastric cancer patients treated with adjuvant (chemo)radiotherapy and/or sugery compared with those with single adjuvant chemotherapy and/or sugery; (4) survival estimates such as OS, DFS or RFS were given. We excluded non-RCTs, reviews, editorials, letters, and trials with no relevant outcomes or sufficient data for analysis. OS was defined as the time from randomisation to death from any cause, and DFS or RFS was defined as the time from randomisation to first reappearance of gastric cancer or death from any cause. We applied 5-year survival statistics in that it is more useful in aggressive diseases that have a shorter life expectancy following diagnosis, such as gastric cancer.

### Data abstraction

Data from the included trials were abstracted using a predefined data abstraction form for each trial containing the following baseline characteristics: the first author name, publication year, trial country, inclusion period, patient mean/median age, tumor stage, median follow up period, total sample size, type of lymphadenectomy, mean/median number of dissected lymph nodes, treatment regimen, total RT dose (Gy) and primary endpoints.

### Assessment of methodological quality

Two independent investigators assessed the methodological quality of each trial included in the present meta-analysis using the tool recommended by the Cochrane Handbook for Systematic Reviews of Interventions for RCTs [26]. Seven domains were assessed, namely, random sequence generation; allocation concealment; blinding of participants, personnel, blinding of outcome assessment; completeness of outcome data; freedom from selective reporting; and freedom from other bias.

### Statistical analysis

The statistical data analysis was performed using Stata^®^ version 12.0 (StataCorp LP, College Station, Texas, USA). The presence of heterogeneity among trials was assessed by using Cochrane’s ^χ2^ test, defining a *p* value less than 0.10 as evidence of heterogeneity. The extent of heterogeneity was assessed using *I*^2^ statistic, with an *I*^2^ more than 50% indicating substantial heterogeneity [27]. We used the random-effect model to combine the effect estimates and subgroup analyses were also applied to further investigate the sources of heterogeneity. We proposed the following study characteristics for heterogeneity including inclusion period, trial region, sample size, treatment regimen, total radiotherapy dose and patient follow-up period. Summary estimates with 95% CIs were obtained for each outcome measure (OS or DFS/PFS). Publication bias was also tested by inspection funnel plot asymmetry and with Begg’s rank correlation test and Egger’s linear regression test, with a *P* value less than 0.05 as an indication of publication bias [28, 29]. Sensitivity analysis using the Duval and Tweedie “trim-and-fill” method has also been conducted to further confirm the robustness of the main analyses [30].

## SUPPLEMENTARY MATERIALS TABLE



## References

[1] Van Cutsem E, Sagaert X, Topal B, Haustermans K, Prenen H (2016). Gastric cancer. Lancet.

[2] Cunningham D, Allum WH, Stenning SP, Thompson JN, Van de Velde CJ, Nicolson M, Scarffe JH, Lofts FJ, Falk SJ, Iveson TJ, Smith DB, Langley RE, Verma M, MAGIC Trial Participants (2006). Perioperative chemotherapy versus surgery alone for resectable gastroesophageal cancer. N Engl J Med.

[3] Park SH, Sohn TS, Lee J, Lim DH, Hong ME, Kim KM, Sohn I, Jung SH, Choi MG, Lee JH, Bae JM, Kim S, Kim ST (2015). Phase III Trial to Compare Adjuvant Chemotherapy With Capecitabine and Cisplatin Versus Concurrent Chemoradiotherapy in Gastric Cancer: Final Report of the Adjuvant Chemoradiotherapy in Stomach Tumors Trial, Including Survival and Subset Analyses. J Clin Oncol.

[4] Zhu WG, Xua DF, Pu J, Zong CD, Li T, Tao GZ, Ji FZ, Zhou XL, Han JH, Wang CS, Yu CH, Yi JG, Su XL, Ding JX (2012). A randomized, controlled, multicenter study comparing intensity-modulated radiotherapy plus concurrent chemotherapy with chemotherapy alone in gastric cancer patients with D2 resection. Radiother Oncol.

[5] Yu C, Yu R, Zhu W, Song Y, Li T (2012). Intensity-modulated radiotherapy combined with chemotherapy for the treatment of gastric cancer patients after standard D1/D2 surgery. J Cancer Res Clin Oncol.

[6] Leong T, Smithers BM, Haustermans K, Michael M, Gebski V, Miller D, Zalcberg J, Boussioutas A, Findlay M, O’Connell RL, Verghis J, Willis D, Kron T (2017). TOPGEAR: A Randomized, Phase III Trial of Perioperative ECF Chemotherapy with or Without Preoperative Chemoradiation for Resectable Gastric Cancer: Interim Results from an International, Intergroup Trial of the AGITG, TROG, EORTC and CCTG. Ann Surg Oncol.

[7] Kim TH, Park SR, Ryu KW, Kim YW, Bae JM, Lee JH, Choi IJ, Kim YJ, Kim DY (2012). Phase 3 trial of postoperative chemotherapy alone versus chemoradiation therapy in stage III-IV gastric cancer treated with R0 gastrectomy and D2 lymph node dissection. Int J Radiat Oncol Biol Phys.

[8] Macdonald JS, Smalley SR, Benedetti J, Hundahl SA, Estes NC, Stemmermann GN, Haller DG, Ajani JA, Gunderson LL, Jessup JM (2001). Chemoradiotherapy after surgery compared with surgery alone for adenocarcinoma of the stomach or gastroesophageal junction. N Engl J Med.

[9] Smalley SR, Benedetti JK, Haller DG, Hundahl SA, Estes NC, Ajani JA, Gunderson LL, Goldman B, Martenson JA, Jessup JM (2012). Updated analysis of SWOG-directed intergroup study 0116: a phase III trial of adjuvant radiochemotherapy versus observation after curative gastric cancer resection. J Clin Oncol.

[10] Lee J, Lim DH, Kim S, Park SH, Park JO, Park YS, Lim HY, Choi MG, Sohn TS, Noh JH, Bae JM, Ahn YC, Sohn I (2012). Phase III trial comparing capecitabine plus cisplatin versus capecitabine plus cisplatin with concurrent capecitabine radiotherapy in completely resected gastric cancer with D2 lymph node dissection: the ARTIST trial. J Clin Oncol.

[11] Bamias A, Karina M, Papakostas P, Kostopoulos I, Bobos M, Vourli G, Samantas E, Christodoulou C, Pentheroudakis G, Pectasides D, Dimopoulos MA, Fountzilas G (2010). A randomized phase iii study of adjuvant platinum/docetaxel chemotherapy with or without radiation therapy in patients with gastric cancer. Cancer Chemother Pharmacol.

[12] Dent DM, Werner ID, Novis B, Cheverton P, Brice P (1979). Prospective randomized trial of combined oncological therapy for gastric carcinoma. Cancer.

[13] Hallissey MT, Dunn JA, Ward LC, Allum WH, British Stomach Cancer Group (1994). The second British Stomach Cancer Group trial of adjuvant radiotherapy or chemotherapy in resectable gastric cancer: five-year follow-up. Lancet.

[14] Kwon HC, Kim MC, Kim KH, Jang JS, Oh SY, Kim SH, Kwon KA, Lee S, Lee HS, Kim HJ (2010). Adjuvant chemoradiation versus chemotherapy in completely resected advanced gastric cancer with D2 nodal dissection. Asia Pac J Clin Oncol.

[15] Moertel CG, Childs DS, O’Fallon JR, Holbrook MA, Schutt AJ, Reitemeier RJ (1984). Combined 5-fluorouracil and radiation therapy as a surgical adjuvant for poor prognosis gastric carcinoma. J Clin Oncol.

[16] Shchepotin IB, Evans SR, Chorny V, Osinsky S, Buras RR, Maligonov P, Shabahang M, Nauta RJ (1994). Intensive preoperative radiotherapy with local hyperthermia for the treatment of gastric carcinoma. Surg Oncol.

[17] Skoropad V, Berdov B, Zagrebin V (2002). Concentrated preoperative radiotherapy for resectable gastric cancer: 20-years follow-up of a randomized trial. J Surg Oncol.

[18] Skoropad VY, Berdov BA, Mardynski YS, Titova LN (2000). A prospective, randomized trial of pre-operative and intraoperative radiotherapy versus surgery alone in resectable gastric cancer. Eur J Surg Oncol.

[19] Stahl M, Walz MK, Stuschke M, Lehmann N, Meyer HJ, Riera-Knorrenschild J, Langer P, Engenhart-Cabillic R, Bitzer M, Königsrainer A, Budach W, Wilke H (2009). Phase III comparison of preoperative chemotherapy compared with chemoradiotherapy in patients with locally advanced adenocarcinoma of the esophagogastric junction. J Clin Oncol.

[20] Zhang ZX, Gu XZ, Yin WB, Huang GJ, Zhang DW, Zhang RG (1998). Randomized clinical trial on the combination of preoperative irradiation and surgery in the treatment of adenocarcinoma of gastric cardia (AGC) —Report on 370 patients. Int J Radiat Oncol Biol Phys.

[21] Zhou ML, Kang M, Li GC, Guo XM, Zhang Z (2016). Postoperative chemoradiotherapy versus chemotherapy for R0 resected gastric cancer with D2 lymph node dissection: An up-to-date meta-analysis. World J Surg Oncol.

[22] Soon YY, Leong CN, Tey JC, Tham IW, Lu JJ (2014). Postoperative chemo-radiotherapy versus chemotherapy for resected gastric cancer: A systematic review and meta-analysis. J Med Imaging Radiat Oncol.

[23] Ohri N, Garg MK, Aparo S, Kaubisch A, Tome W, Kennedy TJ, Kalnicki S, Guha C (2013). Who benefits from adjuvant radiation therapy for gastric cancer? A meta-analysis. Int J Radiat Oncol Biol Phys.

[24] Li LL, Xie CY, Su HF (2014). Benefit of radiotherapy on survival in resectable gastric carcinoma: A meta-analysis. Tumor Biol.

[25] Dai Q, Jiang L, Lin RJ, Wei KK, Gan LL, Deng CH, Guan QL (2015). Adjuvant chemoradiotherapy versus chemotherapy for gastric cancer: A meta-analysis of randomized controlled trials. J Surg Oncol.

[26] Higgins J (2015).

[27] Deeks JJ, Altman DG, Bradburn MJ (2008). Statistical methods for examining heterogeneity and combining results from several studies in meta-analysis. Systematic Reviews in Health Care: Meta-Analysis in Context, Second Edition.

[28] Egger M, Smith GD, Schneider M, Minder C (1997). Bias in meta-analysis detected by a simple, graphical test. BMJ.

[29] Begg CB, Mazumdar M (1994). Operating characteristics of a rank correlation test for publication bias. Biometrics.

[30] Duval S, Tweedie R (2000). Trim and fill: a simple funnel-plot–based method of testing and adjusting for publication bias in meta-analysis. Biometrics.

