# Supplementary material for: Prognostic effect of adjuvant chemoradiotherapy for patients with gastric cancer: an updated evidence of randomized controlled trials
Source: Oncotarget. 2017 Oct 24;8(61):102880–7. doi: 10.18632/oncotarget.21983 (PMC5732696; doi:10.18632/oncotarget.21983)
Supplement: Supplementary file 1 [file oncotarget-08-102880-s001.pdf]

Prognostic effect of adjuvant chemoradiotherapy for patients with gastric cancer: an updated evidence of randomized controlled trials

SUPPLEMENTARY MATERIALS

Search Strategies of the Major Databases

Search date: April 27 2017

Search strategy for Pubmed

1. “Stomach Neoplasms”[Mesh]
2. (gastric OR Stomach)[Title/Abstract]
3. (Cancer OR tumor OR tumour OR carcinoma OR neoplas* OR malignan*) [Title/Abstract]
4. 2 AND 3
5. 1 OR 4
6. “Radiotherapy”[Mesh]
7. (radiotherap* OR radio-therap*)[Title/Abstract]
8. 6 OR 7
9. randomized controlled trial[Publication Type]
10. controlled clinical trial[Publication Type]
11. (randomi?ed OR placebo OR randomly) [Title/Abstract]
12. trial[Title]
13. 9 OR 10 OR 11 OR 12
14. 5 AND 8 AND 13

Results 169

Search strategy for Embase

691

1. ‘stomach tumor’/exp
2. (gastric OR Stomach):ab,ti
3. (Cancer OR tumor OR tumour OR carcinoma OR neoplas* OR malignan*):ab,ti
4. 2 AND 3
5. 1 OR 4
6. ‘radiotherapy’/exp
7. (radiotherap* OR radio-therap*):ab,ti
8. 6 OR 7
9. ‘randomized controlled trial’:it
10. ‘controlled clinical trial’:it
11. (randomised OR randomized OR placebo OR randomly) :ab,ti
12. trial:ti
13. 9 OR 10 OR 11 OR 12
14. 5 AND 8 AND 13

Search strategy for the Cochrane Library

239

1. MeSH descriptor: [Stomach Neoplasms] explode all trees
2. (gastric or Stomach):ti,ab,kw (Word variations have been searched)
3. (Cancer or tumor or tumour or carcinoma or neoplas* or malignan*):ti,ab,kw (Word variations have been searched)
4. 2 AND 3
5. 1 OR 4
6. MeSH descriptor: [Radiotherapy] explode all trees
7. (radiotherap* or radio-therap*):ti,ab,kw (Word variations have been searched)
8. 6 OR 7
9. “randomized controlled trial”:pt (Word variations have been searched)
10. “controlled clinical trial”:pt (Word variations have been searched)
11. (randomised or randomized or placebo or randomly):ti,ab,kw (Word variations have been searched)
12. trial:ti (Word variations have been searched)
13. 9 OR 10 OR 11 OR 12
14. 5 AND 8 AND 13

Supplementary Table 1: Assessment of risk of bias in included trials

First author	Publication year	1) Random sequence generation (selection bias)	2) Allocation concealment (selection bias)	3) Blinding of participants and personnel (performance bias)-	4) Blinding of outcome assessment (detection bias)	5) Incomplete outcome data (attrition bias)-	6) Selective reporting (reporting bias)	7) Other bias-
Yu	2012	unclear	unclear	high risk	high risk	low risk	low risk	low risk
Smalley	2012	unclear	unclear	high risk	high risk	low risk	low risk	low risk
Kim	2012	unclear	unclear	high risk	high risk	low risk	low risk	low risk
Zhu	2012	unclear	unclear	high risk	high risk	low risk	low risk	low risk
Bamias	2010	low risk	unclear	high risk	low risk	low risk	low risk	low risk
Kwon	2010	low risk	unclear	high risk	high risk	low risk	low risk	low risk
Stahl	2009	low risk	unclear	high risk	high risk	low risk	low risk	low risk
Moertel	1984	unclear	unclear	high risk	high risk	low risk	low risk	low risk
Hallissey	1994	unclear	unclear	high risk	high risk	low risk	low risk	low risk
Zhang	1998	low risk	low risk	high risk	high risk	low risk	low risk	low risk
Skoropard	2002	unclear	unclear	high risk	high risk	low risk	low risk	low risk
Skoropard	2000	unclear	unclear	high risk	high risk	low risk	low risk	low risk
Shchepotin	1994	low risk	unclear	high risk	high risk	low risk	low risk	low risk
Dent	1979	low risk	unclear	high risk	low risk	low risk	low risk	low risk
Park	2015	unclear	unclear	high risk	low risk	low risk	low risk	low risk
